# Supplementary material for: Plasma Exchange Reduces Aβ Levels in Plasma and Decreases Amyloid Plaques in the Brain in a Mouse Model of Alzheimer’s Disease
Source: Int J Mol Sci. 2023 Dec 4;24(23):17087. doi: 10.3390/ijms242317087 (PMC10706894; doi:10.3390/ijms242317087)
Supplement: Supplementary file 1 [file ijms-24-17087-s001.zip › ijms-2714177-supplementary.pdf]

Supplementary Figure S1 Whole brain scan, sagittal sections 4G8 immunostaining. Control (a); Plasma exchange (b). Microglia marker Iba1 visualized by immunofluorescence staining, arrowhead shows clusters of Iba1 positive cells (c). Comparison of Iba1 burden ($p = 0.0283$) (d). Astrocyte marker GFAP visualized by immunohistochemistry, arrowhead shows clusters of GFAP positive cells (e). Comparison of the GFAP burden ($p=0.069$). Data were analyzed and plotted as a percentage of control. Graphs represent the mean ± standard error of the mean (SEM).

Supplementary Figure S2 PE reduces the insoluble Aβ fraction in brain ($p = 0.039$) (a). No differences observed in soluble Aβ fraction ($p=0.234$) (b). Graphs represent the mean ± standard error of the mean (SEM).

Supplementary Figure S3 PE delays the progressive increase of Aβ in plasma from 3mo. APP/PS1 throughout the treatment until 7 months old. No significant differences between the plasma collected at different time points. Graphs represent the mean ± standard error of the mean (SEM).

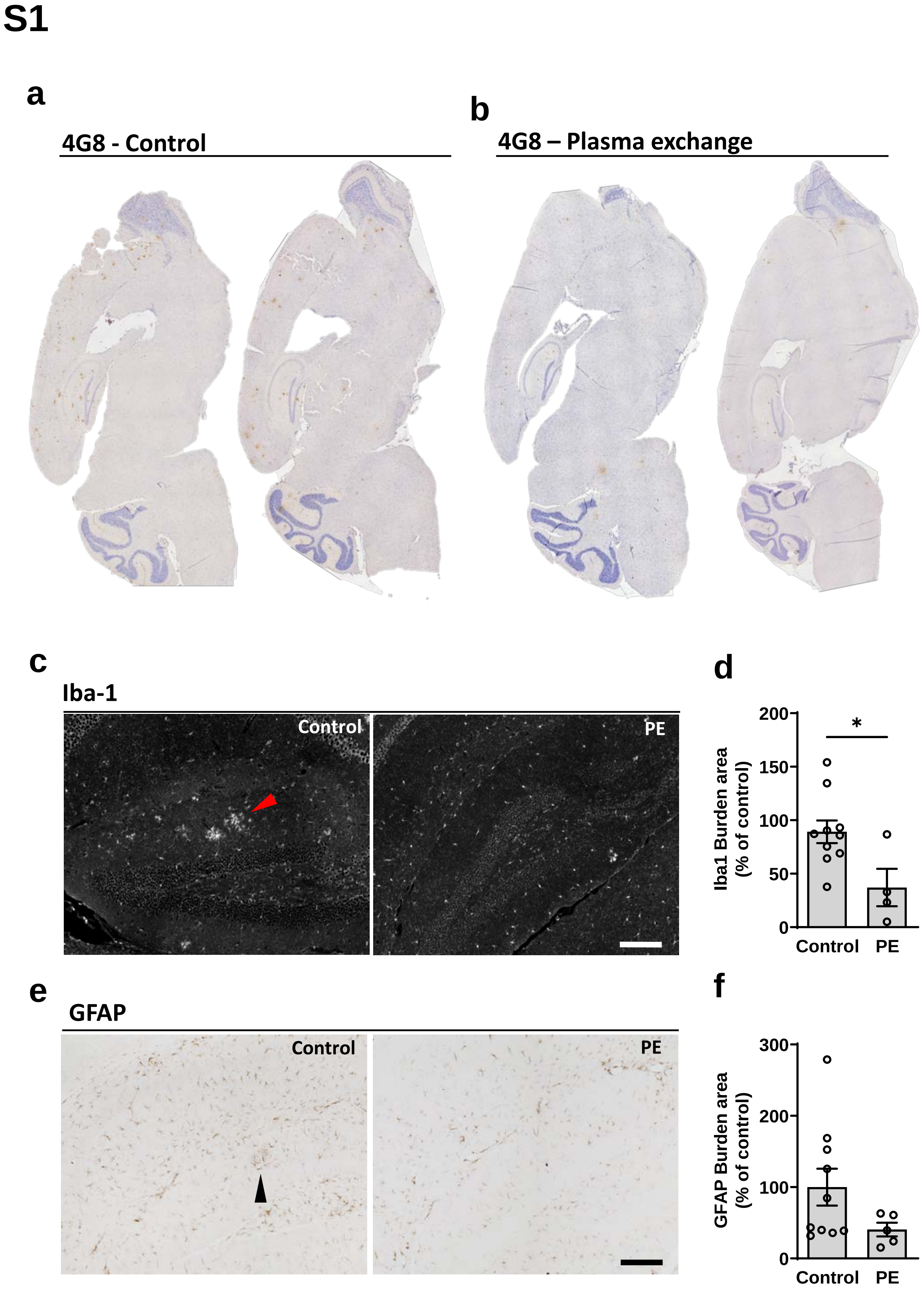

S1
a
4G8 - Control
b
4G8 – Plasma exchange
c
Iba-1
Control
PE
d
Iba1 Burden area
(% of control)
200
150
100
50
0
*
Control
PE
e
GFAP
Control
PE
f
GFAP Burden area
(% of control)
300
200
100
0
Control
PE

S2

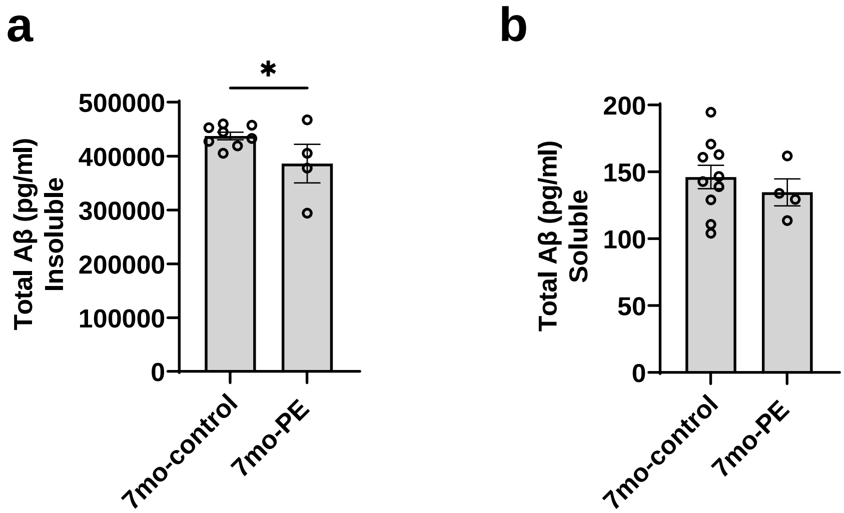
a
b
*
Total Aβ (pg/ml)
Insoluble
500000
400000
300000
200000
100000
0
7mo-control
7mo-PE
Total Aβ (pg/ml)
Soluble
200
150
100
50
0
7mo-control
7mo-PE

S3

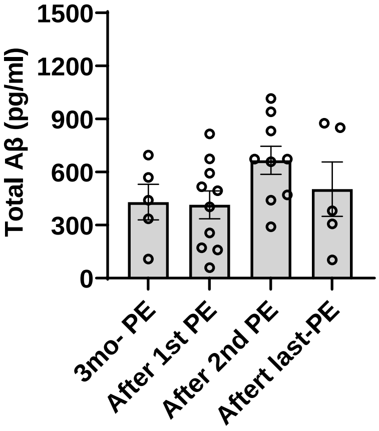
Total Aβ (pg/ml)
1500
1200
900
600
300
0
3mo- PE
After 1st PE
After 2nd PE
Aftert last-PE
